# Caffeine and Headache: Exploring the Multifaceted Relationship

**DOI:** 10.1002/brb3.71527

**Published:** 2026-06-08

**Authors:** Mona Hussein, Amr Hassan, Salsabil Abo Al‐Azayem, Janu Thuraiaiyah, Henrik Winther Schytz, Anter Mothenna Abdullah Nasser, Rehab Magdy

**Affiliations:** ^1^ Department of Neurology Beni‐Suef University Beni‐Suef Egypt; ^2^ Department of Neurology Cairo University Cairo Egypt; ^3^ Danish Headache Center, Department of Neurology, Rigshospitalet– Glostrup, Faculty of Health and Medical Sciences University of Copenhagen Copenhagen Denmark; ^4^ Department of Clinical Medicine, Faculty of Health and Medical Sciences University of Copenhagen Copenhagen Denmark; ^5^ Department of Neurology Thamar University Dhamar Yemen

**Keywords:** adenosine, caffeine, caffeine‐withdrawal headache, headache, migraine

## Abstract

**Purpose:**

To explore the multifaceted relationship between caffeine and headache disorders, focusing on its dual role as both an analgesic and a potential trigger and to summarize the mechanisms underlying its anti‐nociceptive effects.

**Method:**

This narrative review synthesizes evidence from experimental, clinical, and epidemiological studies on caffeine's pharmacological actions, its role in adenosine receptor modulation, and its impact across different headache types, including migraine, hypnic headache, post‐dural puncture headache (PDPH), medication‐overuse headache (MOH), and caffeine‐withdrawal headache.

**Findings:**

Caffeine exerts analgesic effects through adenosine receptor antagonism, prostaglandin inhibition, GABA‐A modulation, and cholinergic facilitation while also enhancing the efficacy of analgesics such as nonsteroidal anti‐inflammatory medications (NSAIDs), acetaminophen, and opioids. In migraine, it demonstrates a dual role, relieving attacks by counteracting adenosine‐mediated vasodilation and improving drug absorption yet potentially triggering them when intake is excessive or inconsistent due to mechanisms such as magnesium depletion, diuresis, and sleep disruption. Beyond migraine, caffeine shows therapeutic benefit in hypnic headache and PDPH, but chronic use may contribute to MOH through neuroadaptive changes. Abrupt cessation of caffeine often provokes caffeine‐withdrawal headache.

**Conclusion:**

Caffeine plays a complex role in headache disorders, acting as both a therapeutic agent and a potential trigger. Its effects depend on dosage, timing, individual susceptibility, and headache subtype. Understanding these mechanisms is essential for guiding clinical recommendations and optimizing caffeine use in headache management.Caffeine plays a complex role in headache disorders, acting as both a therapeutic agent and a potential trigger. In migraine, it demonstrates a dual role, either relieving or triggering. Beyond migraine, caffeine shows therapeutic benefit in hypnic headache, spontaneous intracranial hypotension, and PDPH, but chronic use may exacerbate idiopathic intracranial hypertension and contribute to MOH.

## Graphical abstract

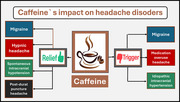

## 1 Introduction

Caffeine is currently the most commonly consumed psychoactive substance, with almost 80% of people consuming a caffeinated product daily. Caffeine is naturally present in various foods, including coffee beans, mate leaves, cocoa beans, tea leaves, chocolate, cola, and nuts. Moreover, various medications, dietary supplements, and soft and energy drinks contain caffeine (Frary et al. 2005; Heckman et al. 2010; Ogawa and Ueki 2007).

Caffeine stimulates wakefulness, increases concentration, and decreases fatigue. However, higher doses may induce headache, anxiety, nervousness, irritability, nausea, poor sleep, tremor, and tachycardia (Grosso et al. 2017; Miners and Birkett 1996; Nawrot et al. 2003).

Caffeine has been reported to have a strong analgesic effect, mainly due to its action on adenosine receptors involved in nociception (Jacobson et al. 2022). There is cumulative evidence that caffeine has adjuvant effects, increasing the analgesic effects of various analgesic medications (Boppana et al. 2022).

Recently, several studies have explored the ambiguous role of caffeine in headache. In migraine, caffeine has been reported to have a dual effect: triggering on one side and relieving on the other side (Alstadhaug and Andreou 2019; Lipton et al. 2017). In post‐dural puncture headache (PDPH) and hypnic headache, caffeine and caffeine‐containing analgesics were found to be effective treatments (Camann et al. 1990; Derry et al. 2014; Liang and Wang 2014). On the other hand, chronic caffeine consumption was implicated in the mechanisms of the generation of medication‐overuse headache (MOH) (Espinosa Jovel and Sobrino Mejía 2017). It has also been shown to increase the risk of chronic daily headache and to cause chronification of migraine (Scher et al. 2004; Zhang et al. 2023). Abrupt cessation of caffeine was reported to cause severe headache, codified as caffeine‐withdrawal headache (“Headache Classification Committee of the International Headache Society (IHS) The International Classification of Headache Disorders, third edition,” 2018).

The underlying physiological mechanisms of such multifaceted effects of caffeine on various headache disorders need further exploration. In this review article, we aimed to delve deeper into caffeine's anti‐nociceptive mechanism and clarify its role in different headache disorders.

## 2 Methods

A comprehensive literature search was conducted across PubMed, Scopus, and Web of Science using combinations of the following keywords: caffeine, headache, migraine, hypnic headache, PDPH, MOH, idiopathic intracranial hypertension, and caffeine withdrawal. The search covered all available literature up to 2025, restricted to English‐language articles. Inclusion criteria comprised experimental, clinical, and epidemiological studies that directly addressed the relationship between caffeine and headache disorders. Exclusion criteria included non‐peer‐reviewed sources, case reports without mechanistic or clinical relevance, and studies focusing on caffeine's effects unrelated to headache. Studies were initially screened by title and abstract, followed by a full‐text review. Priority was given to randomized controlled trials, large observational studies, systematic reviews, and meta‐analyses, while mechanistic and preclinical studies were included when they provided unique insights into pathophysiology.

## 3 Caffeine and Anti‐Nociceptive Mechanism

The most well‐documented molecular function of caffeine is the regulation of adenosinergic signaling, acting as a competitive antagonist at adenosine receptors (Jacobson et al. 2022). Therefore, before we discuss caffeine's role in pain management, we need to understand adenosine.

### 3.1 Adenosine and Adenosine Receptors

Adenosine is a purine nucleoside vital to many cellular and molecular functions, including metabolism, cell signaling, purinergic neuronal signaling, and inflammation (Jacobson and Gao 2006).

**FIGURE 1:**
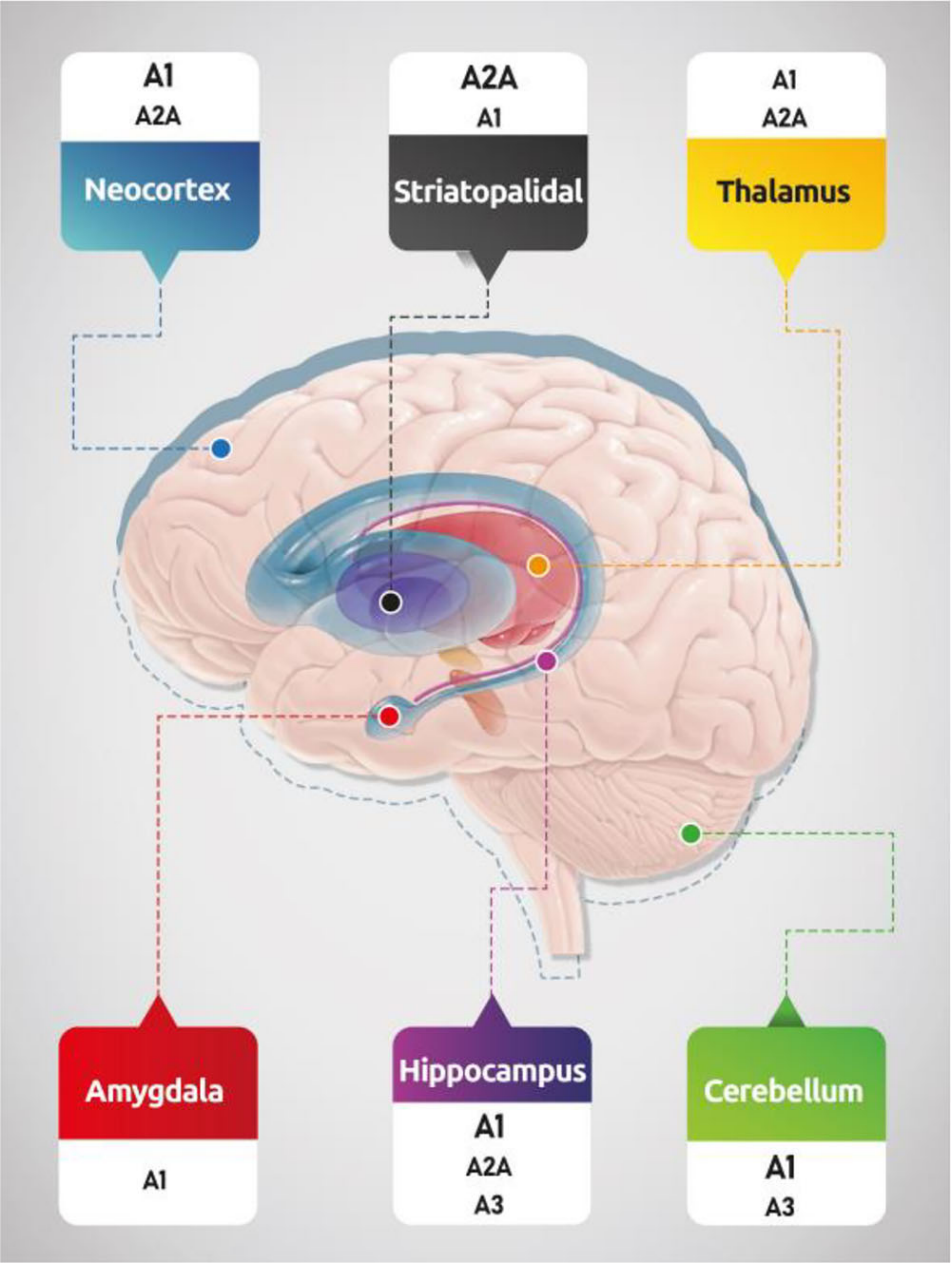
Distribution of adenosine receptors across different brain regions.

Adenosine inhibits neuronal activity in both the central and peripheral nervous systems. There are four types of adenosine receptors found in human bodies (A1, A2A, A2B, and A3) that are expressed in unique parts of the central and peripheral nervous systems (Figure 1) (Sachdeva and Gupta 2013).

Adenosine A1 receptors are the most common adenosine receptors in the brain, and they can be found abundantly in the neocortex, cerebellum, hippocampus, and dorsal horn. Adenosine A2A receptors are more broadly distributed in pre‐ and postsynaptic nerve terminals in the striatum (Sachdeva and Gupta 2013). On the other hand, A2B and A3 receptors are found at low levels in the brain (Borea et al. 2018), with a particular abundance of the latter in the hippocampus and cerebellum (Borea et al. 2017).

### 3.2 Adenosine Signaling

Depending on the anatomical location (central or peripheral), the kind of pain (acute or chronic), and the activated adenosine receptor subtype, adenosine signaling can be either pro‐nociceptive or anti‐nociceptive (Borea et al. 2018; Sheth et al. 2014). Adenosine receptors related to the Gi alpha subunit (A1 and A3) are generally anti‐nociceptive. In contrast, those connected with the Gs alpha subunit (A2A and A2B) are pro‐nociceptive (Fried et al. 2017; Haddad and Cherchi, 2023). According to Sheth et al. (2014), these purinergic G protein‐coupled receptors have distinct effects on adenylate cyclase activity, which enables adenosine to regulate intracellular cAMP levels (Sheth et al. 2014).

### 3.3 Adenosine's Antinociceptive Effect

#### 3.3.1 Inhibition of Cyclic AMP

**FIGURE 2:**
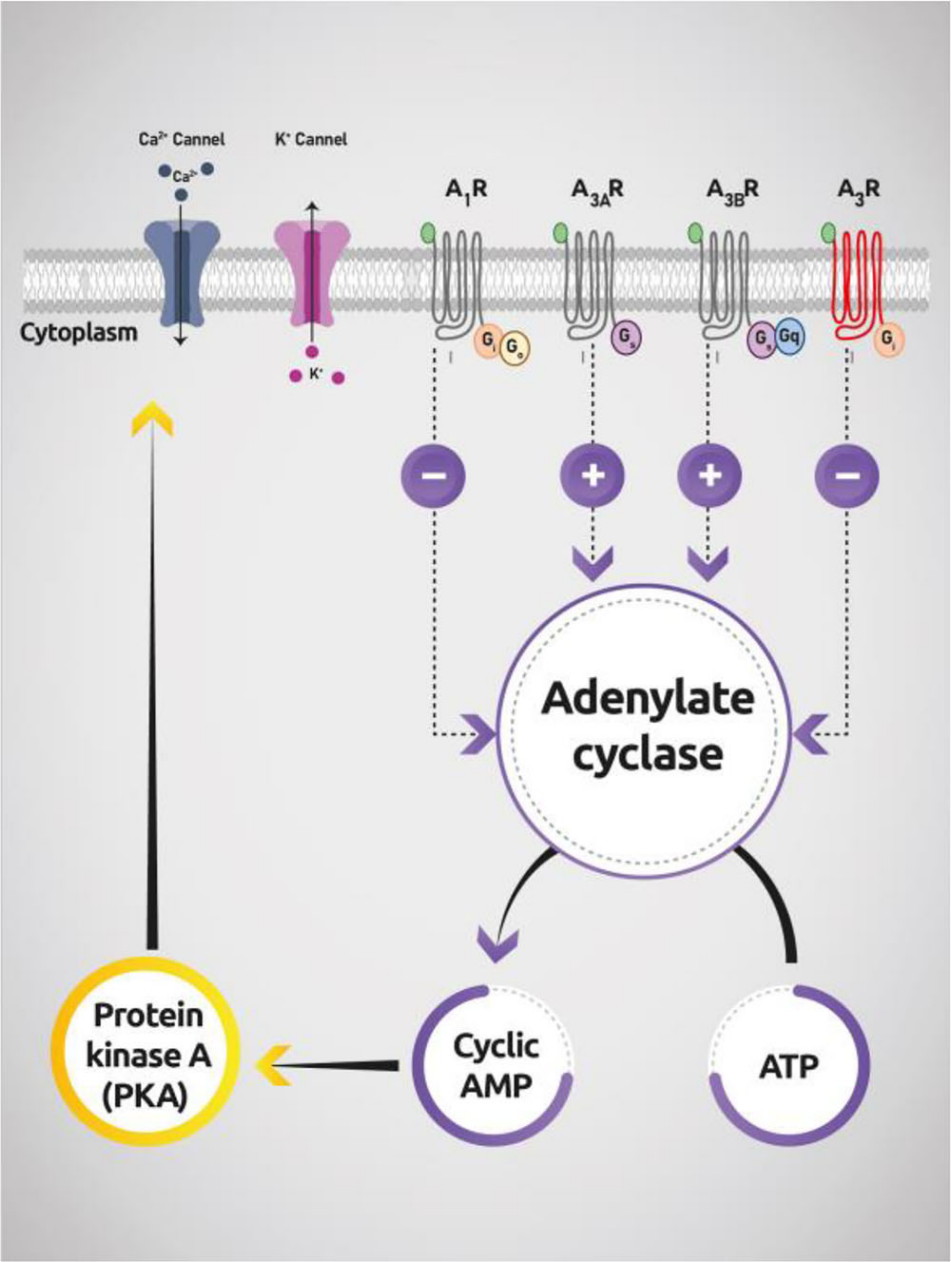
Schematic representation of the four G‐protein coupled adenosine receptors. They have different effects on the activity of adenylate cyclase that are linked to the modulation of Ca and K channels. ATP: Adenosine triphosphate, Cyclic AMP: Cyclic adenosine monophosphate, PKA: Protein kinase A.

Adenosine's antinociceptive impact has been mainly attributed to A1R activation (Vincenzi et al. 2020). Several A1R agonists have been demonstrated to be effective in several preclinical pain models (Thuraiaiyah et al. 2022b; Vincenzi et al. 2023). A1Rs inhibit cyclic AMP, thereby inhibiting the activation of protein kinase A (PKA), which in turn inhibits Ca2+ channels and activates K+ currents, leading to hyperpolarization (Figure 2) (Fried et al. 2017). This receptor subtype is important for analgesic responses because of its unique expression in many pain‐transmission pathways (Fried et al. 2017).

#### 3.3.2 Inhibition of Cyclic Nucleotide Phosphodiesterases (PDEs)

The non‐selective inhibition of PDEs, which raises cytosolic cAMP and subsequently stimulates downstream cAMP‐dependent mechanisms, is another known action of caffeine (Francis et al. 2011; Ialongo et al. 2023).

Moreover, it inhibits cGMP‐specific PDEs, causing vasodilatation in blood vessels by regulating vascular smooth muscle contractility (Echeverri et al. 2010).

#### 3.3.3 Regulating the Activity of GABA‐A Receptors

Another contributing mechanism is the regulation of GABA‐A receptor activity via the benzodiazepine binding site. According to Shi et al. (2003), caffeine at 1 mg significantly inhibited the binding of [3H] diazepam to GABAA receptors in rat cerebral cortical membranes.

#### 3.3.4 Facilitation of Central Cholinergic Transmission

Also, caffeine facilitated central cholinergic transmission by inhibiting acetylcholinesterase, thus promoting central cholinergic analgesia (Ghelardini et al. 1997; Ialongo et al. 2023).

#### 3.3.5 Inhibition of the Synthesis of Prostaglandin E

**FIGURE 3:**
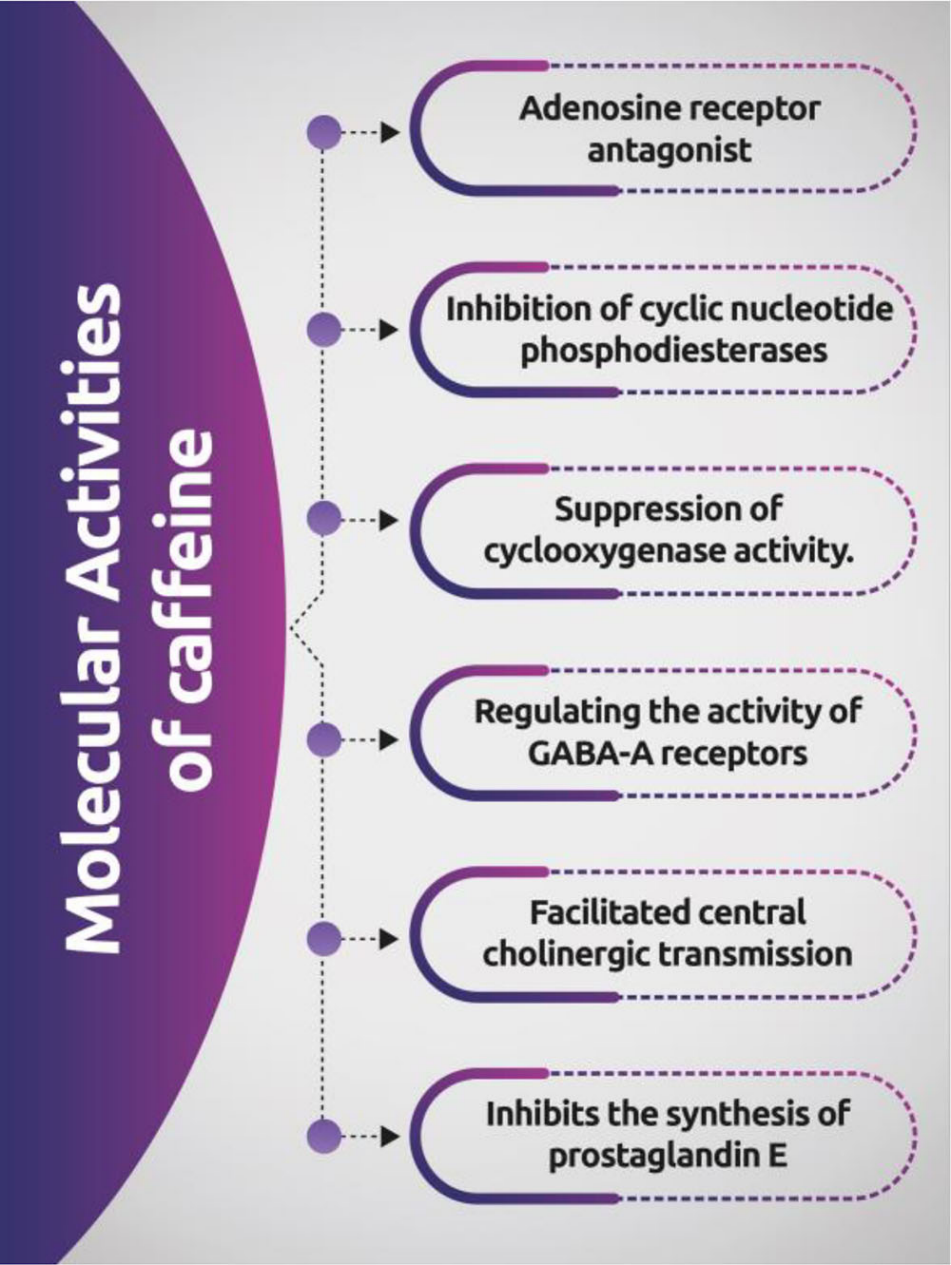
The different mechanisms of the anti‐nociceptive effect of caffeine. GABA‐A: Gamma‐aminobutyric acid type A.

Furthermore, it inhibits prostaglandin E synthesis, which plays a critical central function, including fever generation and pain transmission (Krisnamurti and Fatchiyah, 2019), by suppressing cyclooxygenase‐2 (COX‐2) activity. The latter mechanism can explain the synergistic analgesic effect of caffeine with acetaminophen or acetylsalicylic acid (Fiebich et al. 2000). The different mechanisms of the anti‐nociceptive effect of caffeine are summarized in Figure 3.

## 4 Caffeine as an adjuvant analgesic

Caffeine is utilized as an adjuvant analgesic in a variety of formulations containing nonsteroidal anti‐inflammatory medications (NSAIDs), antidepressants, and acetaminophen (Boppana et al. 2022). It was suggested that caffeine was initially added to simple analgesics to counteract their sedative effects, but it was later found that lower doses of caffeine improved the effects of simple analgesics (Derry et al. 2014; Sawynok 2011). Various clinical trials proved that when caffeine is combined with analgesic drugs, the analgesic dose can be reduced to achieve the same analgesic effect across various types of pain (e.g., headache, postoperative, or postpartum pain) (Ialongo et al. 2023).

Derry et al. (2014) conducted a meta‐analysis of 19 studies involving 7238 individuals. The most common diagnoses of the included individuals were headache and postoperative or postpartum pain. The studies reported adding 100–130 mg of caffeine to simple analgesics (e.g., ibuprofen and paracetamol) and concluded that adding 100 mg or more of caffeine to an analgesic could be worthwhile. Additionally, no significant side effects were reported.

One probable explanation is caffeine's ability to enhance the absorption of NSAIDs or decrease their excretion, thereby increasing their bioavailability (Ialongo et al. 2023; Lipton et al. 2017). An inhibitory effect on A2A and A2B receptors and on COX‐2 activity has been proposed as an explanation for this mechanism (Sawynok 2011; Weiser and Weigmann 2019).

Caffeine also shows beneficial effects when combined with opioids, with numerous studies showing caffeine's adjuvant effect in chronic pain patients receiving opioids (Scott et al. 2017).

The mechanism of this enhancement is not fully understood, but caffeine may increase the release of endogenous opioids, and the involvement of the noradrenergic and adrenergic systems may also contribute to its enhancement of opioid analgesia (Ialongo et al. 2023).

## 5 Caffeine and Migraine

**FIGURE 4:**
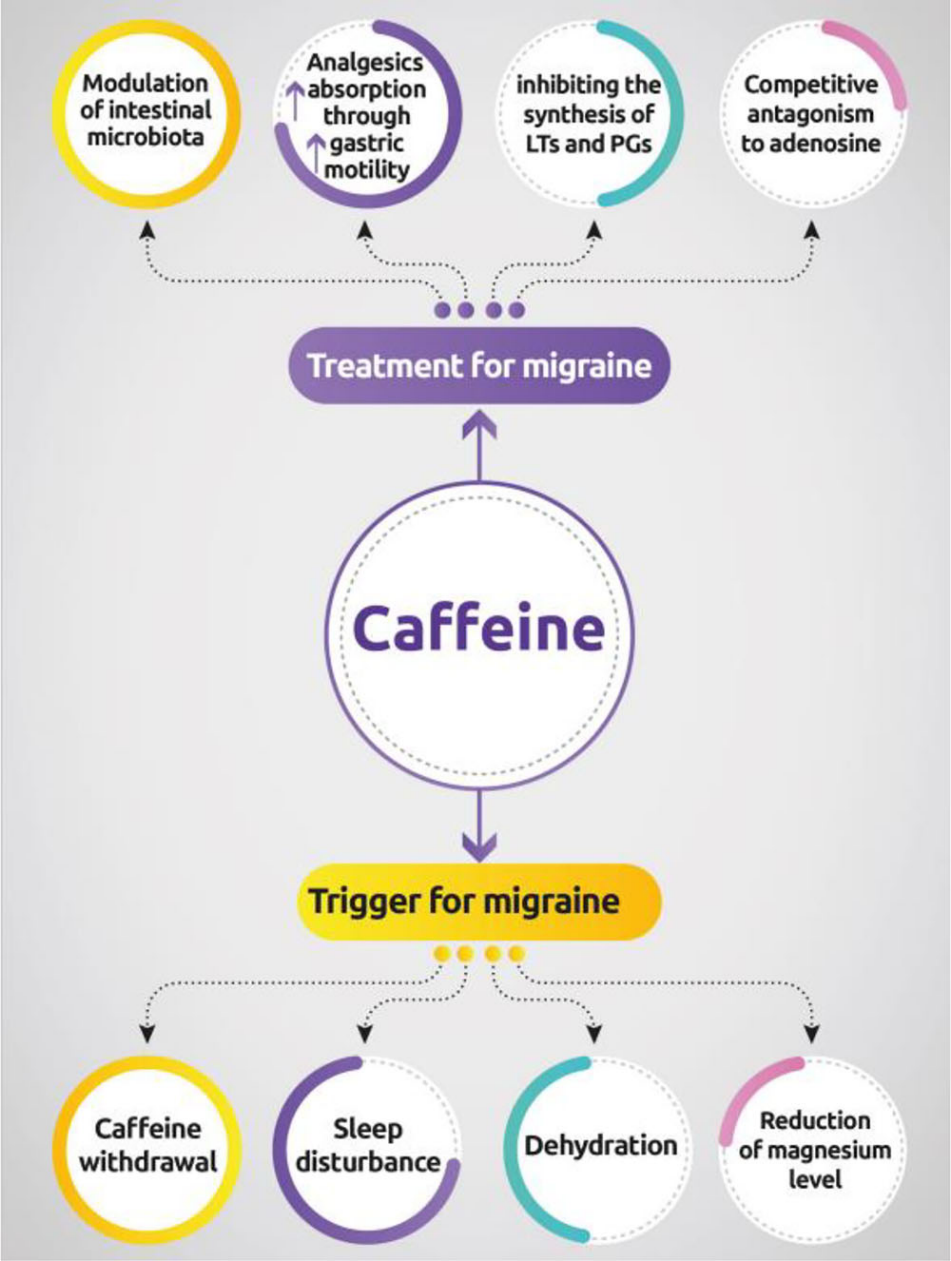
Schematic representation of caffeine's bidirectional effects on migraine. These effects are dose‐dependent, with low‐to‐moderate acute intake potentially exerting migraine‐alleviating effects, whereas high or chronic intake and withdrawal may trigger or exacerbate migraine. The net effect is further modulated by individual variability, including genetic susceptibility, metabolic factors, and lifestyle characteristics.  LTs and PGs: Leukotrienes and Prostaglandins.

The mysterious relationship between caffeine and migraine has sparked the interest of many researchers over the past 20 years. The dual effects of caffeine on migraine, triggering on one side and relieving on the other side, made the relationship between migraine and caffeine a very intriguing topic for migraine researchers. Millions of migraine sufferers who consume caffeine are continuously searching for an answer to the question: Can caffeine be a treatment for migraine attacks, or is it a triggering factor? In other words, is it better for them to consume or avoid caffeine? (Alstadhaug and Andreou 2019; Lipton et al. 2017). The mechanisms by which caffeine can treat or trigger migraine are summarized in Figure 4.

### 5.1 Caffeine as Migraine Treatment

It is well known that adenosine is a neuromodulator with a crucial role in migraine pathophysiology (Thuraiaiyah et al. 2022a; Thuraiaiyah et al. 2022b). Adenosine plasma levels were found to be increased during migraine headache attacks (Guieu, Sampiéri, Bechis, and Rochat 1994), and exogenous adenosine was reported to be a triggering factor for headache attacks (Birk et al. 2005; Thuraiaiyah et al. 2023). Additionally, an adenosine uptake inhibitor (dipyridamole) could induce headache in healthy individuals and increase the frequency and severity of headache attacks in patients with migraine (Guieu et al. 1998; Kruuse et al. 2006). However, it is important to note that dipyridamole also increases cGMP levels; therefore, the observed effects may not be solely attributable to enhanced adenosine signaling but could also involve cGMP‐mediated mechanisms (Hawkes 1978). Since caffeine is known to be a competitive antagonist of adenosine, it may therefore be an effective migraine treatment (Ribeiro and Sebastião 2010).

Convincing evidence suggests that the analgesic mechanism of caffeine in migraine goes beyond its vasoconstrictor effects. Caffeine has been found to inhibit the production of leukotrienes and prostaglandins, which are implicated in migraine pathophysiology (Antonova et al. 2013). Additionally, results from both experimental and clinical studies suggest that caffeine can enhance the analgesic effect of the analgesic drugs when combined with them. This was achieved by increasing gastric motility, thereby increasing the absorption of analgesic drugs (Derry et al. 2014). This effect may provide therapeutic benefits to patients with migraine because they are already known to have gastric stasis not only during migraine attacks but also in between them. Such gastric stasis may slow the absorption of the analgesic drugs, thus reducing their effectiveness (Aurora et al. 2006).

Emerging evidence suggests that caffeine may influence gut micro biota composition, which in turn could affect migraine pathophysiology through the gut‐brain axis. Regular caffeine intake has been associated with shifts in microbial populations, including increases in beneficial taxa such as *Bifidobacterium* and *Prevotella*, alongside reductions in potentially pathogenic species (González et al. 2020). These alterations may modulate immune responses, short‐chain fatty acid production, and neurotransmitter activity (e.g., serotonin and GABA), all of which are implicated in migraine mechanisms (Crawford et al. 2022). Furthermore, micro biota‐driven changes in inflammatory cytokines and purinergic signaling may intersect with adenosine pathways, providing a plausible link between caffeine consumption and migraine modulation (Gazerani et al. 2024). However, current evidence is constrained by small study samples, cross‐sectional designs, and confounding dietary and lifestyle factors, making it difficult to establish causality. It remains unclear whether micro biota changes precede or follow migraine attacks (Gorenshtein et al. 2025). Despite these limitations, the potential therapeutic relevance of micro biota modulation, whether through probiotics, dietary interventions, or controlled caffeine intake, represents a promising but still preliminary therapeutic approach for migraine management that warrants further prospective studies.

### 5.2 Caffeine as Migraine Trigger

It has been reported that there is a nonlinear association between caffeine consumption and the odds of migraine occurrence on that day (Mostofsky et al. 2019). Liao et al. suggested an S‐shaped relationship between caffeine intake and the severity of migraine headache attacks, with an inflection point at roughly 97.5 mg/d. Patients consuming less than 97.5 mg/day had an increased risk of developing severe migraine, whereas in those with an intake ≥97.5 mg/day, no significant association was observed between caffeine consumption and severe migraine (Liao et al. 2025).

Caffeine is known to decrease magnesium levels by reducing its reabsorption (Dolati et al. 2020; Domitrz and Cegielska 2022). The crucial role of magnesium deficiency in the induction of migraine headache attacks has been well‐reported. Also, many researchers emphasize the therapeutic effect of magnesium preparations as a complementary or alternative treatment for the pharmacological treatment of migraine. Therefore, caffeine may be implicated in triggering migraine headache attacks through its effect on magnesium (Dolati et al. 2020; Domitrz and Cegielska 2022).

Caffeine in high doses is well‐known to have an acute diuretic effect (Seal et al. 2017). The impact of daily water intake on the frequency and intensity of migraine attacks has been extensively studied. Dehydration was reported to be one of the possible migraine triggers (Wöber et al. 2007). On the other hand, increased total water intake was significantly associated with a decreased frequency and intensity of migraine headache attacks (Khorsha et al. 2020). So, caffeine in high doses may exacerbate migraine through its diuretic effects (Seal et al. 2017).

There is an evident link between abnormal sleep patterns and increased frequency and intensity of migraine headache attacks. It was observed that migraine attacks increased in the morning after a poor night's sleep or a sleepless night (Brennan et al. 2013). So, the well‐known effect of caffeine on increasing alertness may contribute to triggering migraine attacks (Pasman et al. 2017).

Although caffeine overuse was reported to be one of the modifiable risk factors for migraine chronification (Aguggia and Saracco 2010), caffeine withdrawal was strongly incriminated in increasing the frequency of migraine headache attacks during weekends (Couturier et al. 1992).

Nowaczewska et al. conducted an interesting review that included all articles published up to June 2020 investigating the ambiguous relationship between migraine and caffeine. The main conclusions of their review were: (a) migraine sufferers should not consume more than 200 mg daily and should maintain a constant daily caffeine intake as much as possible to prevent developing caffeine withdrawal headache; (b) migraineurs should preferably consume caffeine at a fixed time each day and should not stop consuming it on weekends; and (c) caffeine‐containing analgesics are an effective therapy for migraine attacks, but their usage should not exceed twice per week to avoid MOH (Nowaczewska et al. 2020).

Notably, terminology in the context of migraine and caffeine requires careful distinction. A trigger refers to a factor that directly precipitates a migraine attack in a susceptible individual (Kelman 2007). A facilitator denotes a condition that lowers the threshold for attacks without necessarily initiating them. A risk factor implies a variable associated with an increased likelihood of migraine occurrence or chronification over time, often identified in epidemiological studies (Scher et al. 2004). An association describes a statistical relationship without evidence of causality. Most studies examining caffeine and migraine are observational and therefore cannot definitively establish causality (Mostofsky et al. 2019). Accordingly, while caffeine intake has been reported to correlate with migraine occurrence, it should be better described as an association or potential facilitator rather than a proven trigger. This distinction is crucial to avoid misinterpretation and to guide future clinical recommendations.

## 6 Caffeine and Hypnic Headache

Hypnic headache is an unusual primary headache disorder that mostly affects patients over the age of 50. Patients usually wake up with a holo‐cranial headache lasting more than 15 min and occurring more than 15 nights per month (“Headache Classification Committee of the International Headache Society (IHS) The International Classification of Headache Disorders, third edition,” 2018). Those patients may experience some migrainous features, such as phono/photo‐phobia and nausea, in addition to some autonomic symptoms such as rhinorrhea and lacrimation (Holle et al. 2010; Melchior et al. 2023).

The underlying pathophysiology of hypnic headache has not yet been conclusively clarified. The pathognomonic circadian rhythmicity of hypnic headache attacks suggests possible hypothalamic involvement (Holle et al. 2014). It has been proposed that hypnic headache may be attributed to melatonin and serotonin dysregulation (Evers and Goadsby 2003). Initially, hypnic headache was thought to be a rapid eye movement (REM) sleep disorder. However, this concept was rejected after the description of non‐REM sleep‐associated hypnic headache attacks (Manni et al. 2004). Some authors suggested that both nocturnal elevation of blood pressure and obstructive sleep apnea may be implicated in the pathogenesis of hypnic headache (Caminero et al. 2010). It is worth declaring that hypnic headache can also occur secondary to structural brain lesions (Garza and Oas 2009; Valentinis et al. 2009).

Due to the rarity of this type of headache, the treatment options for hypnic headache have been based primarily on small case series and expert opinions. These reports revealed that the response of hypnic headache to caffeine is considered one of its pathognomonic features (Holle and Obermann 2012). Caffeine was found to relieve not only hypnic headache attacks but also to be used as a prophylactic compound when given just before sleep (Diener et al. 2012). Caffeine‐containing analgesics also provided substantial relief from hypnic headache attacks (Holle et al. 2010).

Despite the fact that coffee is generally thought to disrupt sleep, its effectiveness in treating hypnic headache probably reflects mechanisms other than wake promotion (Holle and Obermann 2012). Caffeine, as an adenosine receptor antagonist, may counteract abnormal adenosinergic activity during sleep, which has been hypothesized to contribute to the onset of hypnic headache attacks. By blocking adenosine receptors, caffeine may stabilize neuronal activity in sleep‐related brain regions and prevent the cascade leading to hypnic headache. (Evers and Goadsby 2003; Huang et al. 2011) Interestingly, in many cases, patients report that consuming caffeine before bedtime or during an attack can abort or prevent the headache without significantly impairing overall sleep quality. This suggests that the therapeutic mechanism is not simply related to wake promotion, but rather to modulation of specific neurochemical pathways involved in hypnic headache generation (Holle et al. 2014).

## 7 Caffeine and Post‐Dural Puncture Headache (PDPH)

It is well known that PDPH is the most prevalent side effect of lumbar puncture and spinal anaesthesia (Shahriari et al. 2021). The lowering of the pain score in the supine position, while its increase in the upright position, is the characteristic feature of PDPH (Bezov et al. 2010).

PDPH results from cerebrospinal fluid leakage into the epidural space (Barati‐Boldaji et al. 2023). Thus, dural puncture causes excessive CSF leakage, decreased CSF volume, and intracranial hypotension. A decrease in CSF pressure induces strain on the dura and intracerebral structures, leading to PDPH (Dabas et al. 2019). The Monro‐Kellie hypothesis provides another aspect of PDPH pathology. Because the sum of the brain, CSF, and intracranial circulation volumes remains stable, a decrease in CSF volume caused by hypotension during spinal anesthesia triggers compensatory cerebral vasodilation, which may result in pain (Dabas et al. 2019).

Caffeine is among the medications used to treat PDPH (Basurto Ona, Uriona Tuma, Martínez García, Solà, and Bonfill Cosp 2013; Shahriari et al. 2021), before an epidural blood patch may be applied (Sandesc et al. 2005). However, studies have investigated the use of caffeine to treat PDPH with mixed results (Astorino et al. 2011; Baratloo et al. 2016; Halker et al. 2007). In the trial reported by Yücel et al. (1999), participants were randomly assigned to take either 1000 mL of normal saline with 500 mg of caffeine sodium benzoate or 1000 mL of normal saline during the first 90 min following spinal anesthesia. They stated that administering IV caffeine‐sodium benzoate could reduce the incidence of PDPH.

The analgesic mechanism of caffeine in PDPH may be attributed to adenosine receptor blockade, leading to cerebral vasoconstriction and decreased cerebral blood flow and brain blood volume. Caffeine may influence CSF production by modulating sodium‐potassium pump activity in the choroid plexus, possibly through its effects on adenosine receptors or intracellular signaling pathways (Aly and Elazeem 2019; Straube et al. 2004). All these mechanisms most likely relieve PDPH.

## 8 Caffeine and Spontaneous Intracranial Hypotension (SIH)

**FIGURE 5:**
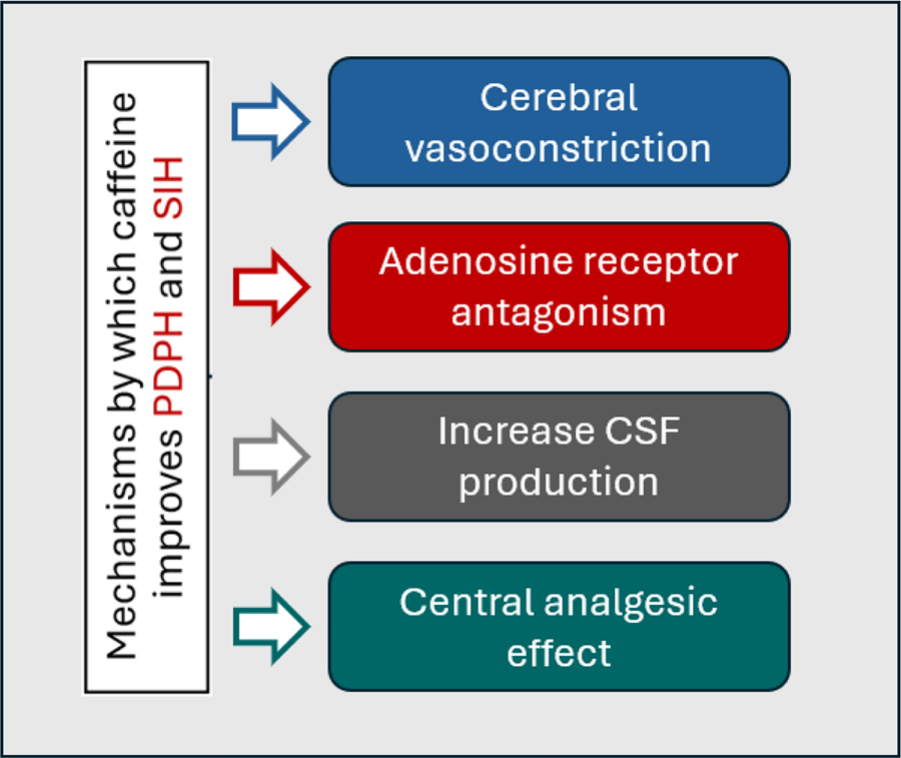
Mechanisms by which caffeine improves PDPH and SIH. PDPH: Post‐dural puncture headache, SIH: Spontaneous intracranial hypotension.

Caffeine plays a supportive, primarily symptomatic role in the management of SIH, a condition typically caused by spinal CSF leaks leading to reduced intracranial pressure (Rau and Cutsforth‐Gregory 2025). The evidence supporting the benefits of caffeine in SIH is largely extrapolated from its established efficacy in PDPH. Caffeine exerts its effects mainly through antagonism of adenosine receptors, cerebral vasoconstriction, increased in CSF production, and a central analgesic effect (Mehta et al. 2023) (Figure 5). These mechanisms may help counteract the compensatory cerebral vasodilation and reduced CSF volume seen in SIH, thereby alleviating hallmark symptoms such as orthostatic headache (García‐Ull et al. 2025).

Clinically, caffeine is often used as an initial conservative measure in mild or early cases, typically administered orally at a dose of 200–300 mg, two to three times daily (Ciceri et al. 2026; Mokri 2015). Despite its widespread use, the therapeutic benefit of caffeine in SIH is generally considered temporary. While some patients report short‐term relief, especially in headache severity, caffeine does not address the underlying structural defect responsible for CSF leakage (García‐Ull et al. 2025). Consequently, its use is often adjunctive, bridging patients until more definitive treatments, such as epidural blood patching, are implemented (Ciceri et al. 2026).

## 9 Caffeine and Medication Overuse Headache (MOH)

Although caffeine‐containing combination analgesics have been implicated in MOH, the mechanisms by which caffeine may contribute to MOH remain incompletely understood (Boppana et al. 2022). Current evidence suggests that MOH is primarily driven by functional neuroplastic changes, including cortical hyperexcitability and enhanced central and peripheral sensitization, rather than established irreversible structural brain damage (Srikiatkhachorn et al. 2014).

Chronic caffeine consumption is known to induce adaptive neurofunctional changes through prolonged antagonism of adenosine receptors, resulting in compensatory upregulation and increased functional sensitivity of adenosine A1 and A2A receptors (Dunwiddie and Masino 2001; Fried et al. 2017). These adaptations may facilitate excitatory neurotransmission, particularly via glutamatergic pathways, and reduce inhibitory modulation, thereby promoting cortical hyperexcitability; one of the key mechanisms implicated in headache chronification and MOH (Srikiatkhachorn et al. 2014).

From an epidemiological perspective, population‐based studies have affirmed a relationship between high habitual caffeine intake and chronic daily headache, supporting a potential link between long‐term caffeine use and headache persistence. However, these observations cannot establish causality and may be influenced by reverse causation, whereby increased caffeine consumption reflects attempts at symptom relief (Espinosa Jovel and Sobrino Mejía 2017; Scher et al. 2004).

Taken together, available evidence suggests that chronic caffeine consumption may contribute to functional neuroadaptive changes that facilitate the development of MOH, particularly in vulnerable individuals. Nevertheless, direct longitudinal or neuroimaging evidence demonstrating caffeine‐induced structural brain alterations leading to MOH is currently lacking, and further mechanistic and prospective studies are required to clarify this relationship.

## 10 Caffeine and Idiopathic Intracranial Hypertension (IIH)

The relationship between IIH and caffeine had not been previously explored until the work conducted by Abdelghaffar et al. (2025). This study provided novel evidence demonstrating, for the first time, that heavy caffeine consumption (≥400 mg/day) in IIH patients is strongly associated with a significantly worse clinical profile. Compared to light or non‐consumers, heavy caffeine users exhibited higher intracranial pressure, worse headache burden, and more severe papilledema, requiring higher acetazolamide doses. Importantly, the study also revealed that excessive caffeine intake was linked to poorer quality of life, increased psychiatric burden (depression, anxiety, stress), and more pronounced sleep disturbances. Taken together, these findings offer a comprehensive perspective suggesting that reducing caffeine intake may represent a promising modifiable lifestyle intervention in the management of IIH (Abdelghaffar et al. 2025).

## 11 Caffeine‐Withdrawal Headache

Headache is a common symptom of abrupt caffeine cessation. Other withdrawal symptoms include fatigue, brain fog, depressed mood, anxiety, irritability, and tremors (Watson 2003). All these symptoms can be explained by the predominant adenosine receptor sites, as presented in Figure 2. Caffeine‐withdrawal syndrome is a clinical diagnosis listed in the Diagnostic and Statistical Manual of Mental Disorders, fifth ed. (DSM–5) (American Psychiatric Association 2013).

The International Classification of Headache Disorders‐3rd edition (ICHD‐3) (Olesen et al. 2018) set the diagnostic criteria for caffeine‐withdrawal headache as headache emerging within 24 h of caffeine cessation in people who used to consume caffeine regularly (> 200 mg/day for more than two weeks). Causality was evident by the development of headache within 24 h of the last caffeine intake and headache relief either within 1 h with 100 mg of caffeine or within 7 days after total caffeine withdrawal.

According to a systematic review conducted by Juliano and Griffiths, the estimated prevalence of caffeine‐withdrawal headache was 24–56% (Juliano and Griffiths 2004). On the other hand, Magdy et al. (2023) investigated caffeine‐withdrawal headache among 755 Muslim caffeine consumers on the first day of Ramadan fasting, during which they prioritized obligatory full abstinence from food and drinks from sunrise to sunset. In this study, 55.5% of participants experienced caffeine‐withdrawal headache. However, these proportions should be interpreted cautiously, as the authors couldn't rule out potential confounders of headaches on the first day of Ramadan, such as fasting‐induced dehydration or hypoglycemia. Nevertheless, all participants in this study were non‐smokers and had never been diagnosed with pre‐existing primary headache disorders.

The vascular mechanism is the most broadly acknowledged mechanism of caffeine‐withdrawal headache. It was found that abrupt caffeine cessation increases cerebral blood flow velocity through enhanced adenosine‐mediated vasodilation (Jones et al. 2000; Sigmon et al. 2009).

Caffeine‐withdrawal headache is predominantly diffuse over the cranium. However, about one‐quarter reported occipital headache (Magdy et al. 2023). This might be attributed to the diffuse distribution of adenosine A1 receptors across the cerebral cortex, with abundant expression in the occipital cortex (Elmenhorst et al. 2012). Phonophobia is reported to be associated with caffeine‐withdrawal headache in about 56.3% of cases (Magdy et al. 2023), which could be explained by the amplified impact of adenosine signaling on cochlear blood flow (Vlajkovic et al. 2009).

The European Food Safety Authority (EFSA) (Efsa Panel on Dietetic Products and Allergies 2015) recommends measuring daily caffeine consumption equated to body weight rather than the absolute quantity of daily consumed caffeine because of the different rates of caffeine metabolism among obese people than others with normal body weight (Lee et al. 2019). Accordingly, a daily caffeine intake/kg body weight of about 2 mg/kg was proposed in Magdy et al. (2023) to outline the definition of caffeine‐withdrawal headache.

**FIGURE 6:**
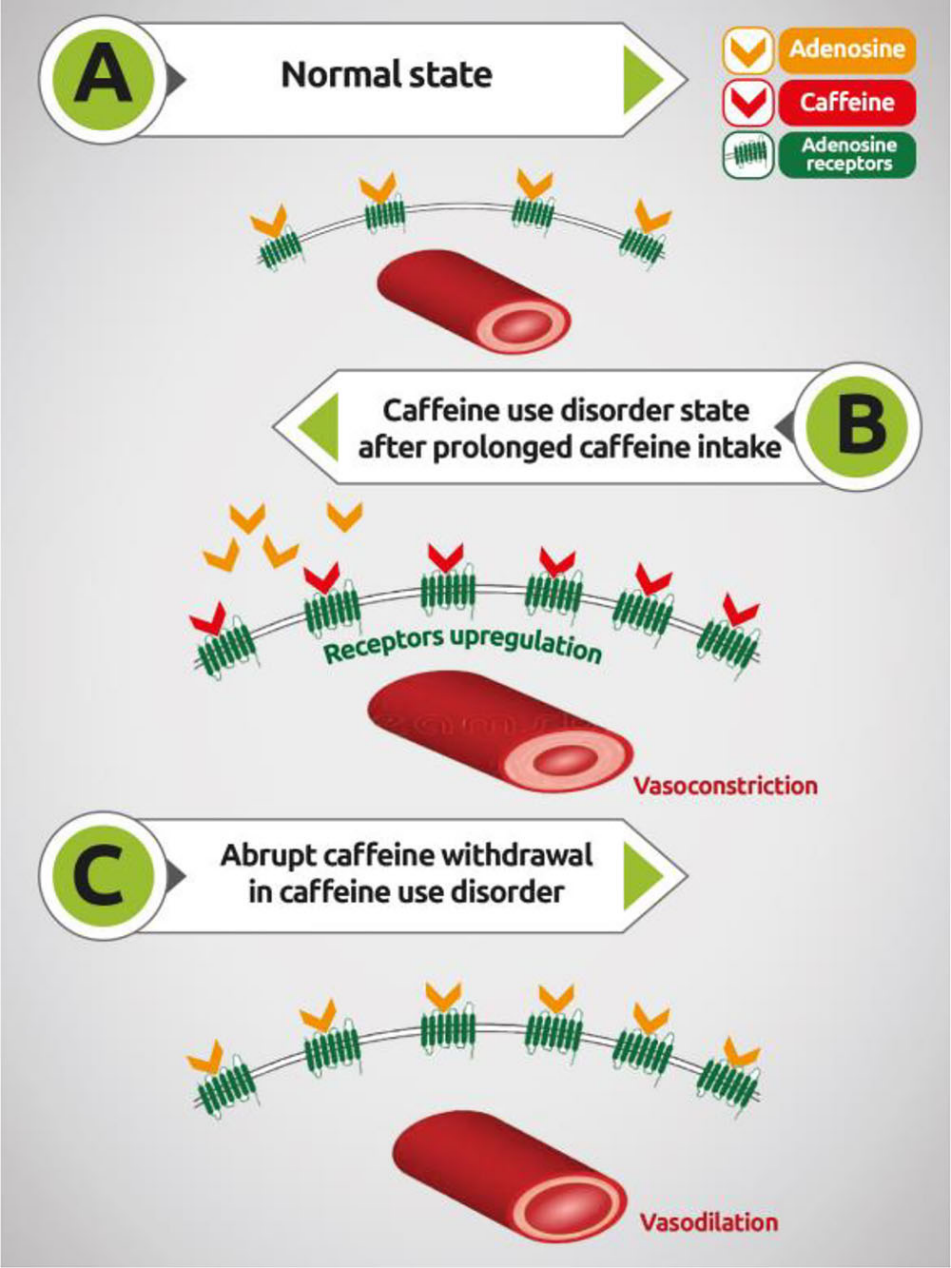
Demonstration of adenosine receptor expression and cerebral vasogenic tone in the normal state (A), in a state of caffeine use disorder (B), and after caffeine withdrawal (C).

Another factor that may account for the probability of occurrence of caffeine‐withdrawal headache is the caffeine use disorder (CUD) and its severity, if present, reflecting how much the individual is physically dependent on regular caffeine intake after long‐term caffeine abuse. People with severe CUD have up regulation of adenosine receptors and augmented functional sensitivity to endogenous adenosine (Fried et al. 2017). Therefore, they would experience intense vasodilation due to a desire‐driven increase in adenosine binding after abruptly refraining from caffeine (Figure 6). The severity of CUDs was found to increase the odds of the occurrence of caffeine‐withdrawal headache by Magdy et al. (2023).

## 12 Clinical Implications and Practical Recommendations

From a clinical perspective, the complex and bidirectional effects of caffeine on headache disorder necessitate an individualized approach. Migraine sufferers should not consume more than 200 mg daily to avoid migraine chronification. Maintaining a consistent daily intake in those patients may be preferable to intermittent or excessive consumption, as fluctuations and abrupt cessation are more likely to precipitate headaches. Complete avoidance is not universally required and should be tailored based on individual trigger patterns. Caffeine consumption above 400 mg/day has been associated with a significantly worse clinical profile in patients with IIH. Caffeine‐containing analgesics can be effective as acute treatment options; however, their use should not exceed twice per week to reduce the risk of MOH. Clinicians should also be aware of caffeine‐withdrawal headache, particularly in habitual consumers, and should recommend gradual dose reduction rather than abrupt cessation. Overall, careful assessment of intake patterns, patient education, and individualized recommendations remain key to optimizing outcomes.

## 13 Conclusion

Caffeine exhibits a complex interplay with the pathophysiologies of headache disorders. In migraine, it can be either a trigger or a treatment based on the total daily amount of caffeine and the consistency of receiving this amount at a fixed time. Caffeine can cause significant relief to both PDPH and hypnic headache, but its regular use can induce structural and functional alterations that may result in MOH. Abrupt cessation of prolonged regular caffeine intake can result in caffeine withdrawal headache due to enhanced adenosine‐mediated vasodilation.

## Author Contributions

MH participated in collecting scientific material, writing, and helped to draft the manuscript. AH participated in collecting scientific material, writing, and helped to draft the manuscript. SA participated in collecting scientific material, writing, and helped to draft the manuscript. JT revised the final manuscript. HS revised the final manuscript. AN revised the final manuscript. RM participated in collecting scientific material, writing, and helped to draft the manuscript. All authors read and approved the final manuscript.

## Funding

The authors have nothing to report.

## Ethics Statement

The authors have nothing to report.

## Consent

The authors have nothing to report.

## Conflicts of Interest

The authors declare no conflicts of interest.

## Declarations

All authors have read and approved the submitted manuscript; the manuscript has not been submitted nor published elsewhere in whole or in part.

## Data Availability

Authors report that the datasets used and/or analyzed during the current study are available from the corresponding author on reasonable request.
